# The Positive Impacts of Exhibition-Driven Tourism on Sustainable Tourism, Economics, and Population: The Case of the Echigo–Tsumari Art Triennale in Japan

**DOI:** 10.3390/ijerph17051489

**Published:** 2020-02-26

**Authors:** Gangwei Cai, Lei Xu, Weijun Gao, Yan Hong, Xiaoyu Ying, Yan Wang, Fanyue Qian

**Affiliations:** 1 College of Civil Engineering and Architecture, Zhejiang University, Hangzhou 310000, China; 11712014@zju.edu.cn; 2 Faculty of Environmental Engineering, University of Kitakyushu, Fukuoka 8080135, Japan; qianfanyue91@163.com; 3 School of Civil Engineering and Architecture, Zhejiang Sci-Tech University, Hangzhou 310000, China; hy@zstu.edu.cn; 4 Department of Architecture, Zhejiang University City College, Hangzhou 310000, China; yingxiaoyu@zucc.edu.cn; 5 School of Architecture, Tianjin Chengjian University, Tianjin 300384, China; wang.y@tcu.edu.cn

**Keywords:** exhibition-driven tourism, sustainable tourism, economic, population, Echigo–Tsumari Art Triennale, empirical analysis

## Abstract

After the recession in Japan in the 1990s, Japanese art exhibitions began to appear. The purpose of these exhibitions was to revitalize these areas through the presentation of art (attracting visitors and tourists). Correspondingly, this study explores the significance of exhibition-driven tourism in Japan. The Echigo–Tsumari Art Triennial (ETAT) was used as a case to study how exhibition-driven tourism has impacted sustainable tourism, economics, and the population. The current paper collected panel data from 1900 to 2018. These panel data were analyzed by descriptive statistics and a correlation analysis (a one-way ANOVA and a Pearson correlation analysis in SPSS26). The empirical analysis showed that the Echigo–Tsumari Art Triennial (exhibition-driven tourism) had positive impacts on sustainable tourism, economics, and the population; its correlations with Niigata were also clear. This study generated results that are valuable from both academic and industry perspectives (exhibition-driven tourism), as this field has not been extensively researched. The current paper also presents the theoretical and practical implications of the statistical results.

## 1. Introduction

### 1.1. Motivation 

From 1961 to 2019, Japan art exhibitions appeared one after another. After the recession in Japan during the 1990s, the number of Japan art exhibitions began to increase precipitously. The number of these exhibitions has particularly exploded since 2007. The purpose of these many exhibitions is to revitalize their host areas (especially in sustainability) through art [1]. Thus, an important question is raised by this article: Did these art exhibitions have any positive sustainable impacts on their hosting areas? 

Among these art exhibitions, the Echigo–Tsumari Art Triennial (ETAT) was chosen as the present case. Triennale means that the exhibition is held once every three years [2]. The ETAT is one of the earliest and most important art exhibitions in Japan [3], as the ETAT has also had a big influence outside of Japan. The ETAT is also one of the largest art festivals in the world [4]. From 2000 to 2018, compelled by this exhibition (the ETAT), more than 2,640,126 people travelled to the host areas. The ETAT has generated exhibition-driven tourism income of more than 57,106 million yen over the past 20 years [5]. 

### 1.2. The Logical Model and Purpose

One of the most famous global examples of the influence of exhibitions is Yiwu, China [6]. The strategy of ‘exhibition-driven trade’ (the Yiwu model of China) has significantly encouraged the joint growth of both exhibitions and trade [7]. These exhibitions may, however, exert both positive and negative impacts. The current paper proposes a relationship between sustainable tourism, economics, the population, and exhibition-driven tourism according to the following theoretical and empirical backgrounds. 

First, previous studies have suggested that temporary exhibitions of modern art have a positive impact on tourism [8]. Therefore, if we want to quantitatively analyze the impact of exhibitions on sustainable tourism (i.e., exhibition-driven tourism), then the number of tourists is a clear and direct indicator [9,10,11]. Second, the tourism industry has emerged as a key force for sustainable (socioeconomic) development globally [12]. Throughout the world, tourism and the travel sector are important economic areas [13]. According to the previous studies by Hwang and his team, local people can have positive impacts on tourism (tourist destination loyalty; sustainable behaviors; eco-friendly behaviors, etc.) [14,15,16]. Thus, the corresponding sustainable economics pertain to the per capita income of the local people. Third, economic changes affect the population to some extent. There are also many theories that attempt to explain how economic growth and rising income levels affect fertility. Theories in this area include the children cost–benefit theories proposed and developed by H. Leibenstein and Gary S. Becker [17]. However, this is a complicated problem. Japan’s population is in decline (especially in rural areas like the hosting areas of the ETAT) [18,19]. Therefore, the corresponding index in this paper uses the number of families as an indicator (although many factors could influence the number of families) [20,21].

**Figure 1:**
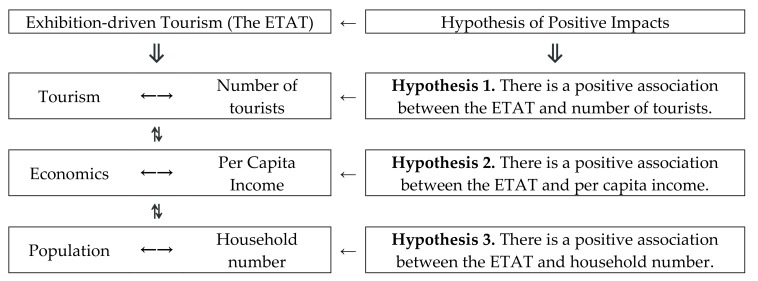
The logical model.

Based on the above parameters, these three aspects (sustainable tourism, economics, and the population) and their corresponding data (number of tourists, per capita income, and household number) are used for the empirical research in this paper (Figure 1). In this way, the current study attempts to fill the related research gap by empirically investigating the correlation between sustainable tourism, economics, population, and exhibition-driven tourism.

The purpose of this research is to study the correlations between sustainable tourism, economics, population, and exhibition-driven tourism. Descriptive statistics and a correlation analysis (a one-way ANOVA and a Pearson correlation analysis in SPSS26) were performed on the three hypotheses (Figure 1) and their panel data [22]. At the same time, the conclusions of this paper provide some positive support and show the impact of Japanese art exhibitions. The hosting areas can achieve their goal of sustainable development through the direct impact of tourism via exhibition-driven tourism. The present study successfully fills this void (the correlation between sustainable tourism, economics, population, and exhibition-driven tourism).

This article contains seven sections. Section 1 is the introduction of the study. Section 2 contains the literature review. Section 3 describes the ETAT. Section 4 provided the research methods of the study. Section 5 is the results section. Section 6 comprises the impacts, limitations, and future research directions. The final section includes the conclusions. 

## 2. Literature Review

“Exhibition-driven” means that something is influenced by an exhibition [5]; this phenomenon has been studied by many scholars. Mu et al. [6] and Wang et al. [7] showed the ‘exhibition-driven trade’ of the Yiwu model. Heald [23] worked in an exhibition-driven environment. Wardani [24] developed a place for art archives, an exhibition-driven artist-run-space. Bunning et al. [25] studied the development of a temporary exhibition-driven impact on the core offerings of the museum. However, there are few direct tourism studies on the influence of exhibitions in previous literature. Since this field has not been extensively researched, the present study generated results that are valuable from both academic and industry perspectives.

### 2.1. Exhibitions and Sustainable Tourism 

First, the idea behind sustainable tourism is to visit locations without harming the local community and nature, and also to have a constructive impact on the environment, society, and economy of the country [26]. Han and Hwang [14] studied the value-belief-emotion-norm model to promote customers’ eco-friendly behavior. They also made a meaningful contribution to advancing eco-friendly activities (sustainable behaviors) for the sake of environmental protection behaviors [15]. There is a consensus that tourism growth should be sustainable, although the question of how to achieve this remains a subject of debate [27]. Tourism can include transportation to the general area, local transportation, accommodations, leisure, entertainment, shopping, and nourishment [28]. The features of sustainable urban tourism and factors of tourism shopping have been previously explored [29]. 

Second, the current paper proposes a relationship between exhibition mechanisms and tourism brand effects [30,31,32]. Kanwel et al. [33] examined the impact of destination image on tourists’ loyalty and intention to visit in Pakistan. Han and Hwang investigated international medical travelers’ post-purchase decision-making process by utilizing key concepts in medical tourism (e.g., first-time vs. repeat experiences) [21]. The present study also proposes a relationship between healthcare and tourism [11]. Medical tourism is largely believed to be a service that combines tourism activities (even exhibition-driven medical tourism) with medical services [34].

Third, as per the report of the WTO (World Tourism Organization) in 2018, international tourists spent $1.3 billion per day and a total of $462 billion in the year 2001 alone [35]. The cultural amenities of a destination, such as museums, monuments, and art exhibitions, provide some of the main attractions for cultural tourists. Most research has focused on tourism demand and the influence of exchange rate and income on tourism revenue [36]. Others have studied political impacts and used time series analyses [37,38].

### 2.2. Exhibitions and Economics 

First, the tourism industry remains an important source for the generation of income in formal and informal sectors in many countries [39]. Hwang and Lee [20] claimed that economic growth and development is rapidly increasing in Korea due to a surge in elderly tourism. This increase shows that tourists feel inner satisfaction, which positively affects their future behavioral intentions [40]. 

Second, sustainable economic growth involves economic development that attempts to satisfy the needs of humans but in a manner that sustains natural resources and the environment for future generations [11,35]. Vasylieva et al. [41] investigated the relationships between the economic, social, and environmental dimensions of sustainable development. Wang et al. [42] studied the theory of the circular economy and the problems existing in the development of the green exhibition in China. Azam and Sarker [43] explored the green tourism in the context of climate change towards sustainable economic development in the south Asian region. Wang et al. [44] analyzed the influence of the exhibition industry on the ecological environment and proposed countermeasures.

Third, many previous studies have empirically supported the influence of conventions and exhibitions on economic growth. One of the earliest studies was an economic impact assessment of the tall mast sailing ceremony of Rhode Island by Della et al. in 1977 [45]. Dwyer et al. [46] believed that the impacts of conventions and exhibitions on the local economy are usually the largest. The selection of evaluation objects for empirical research usually focuses on specific exhibition activities and the overall exhibition industry. Research on the economic impacts of the overall exhibition industry in a specific area includes a study by Kim et al. [29]. Litvin et al. [47] used a case study to describe the ’rising tide’, which refers to the economic contribution from the increased hotel occupancy rate during a festival. Rephann [46] studied the impact assessment of economic activities during the construction and operational periods of exhibition venues. An event may significantly increase local economic activity, but the net impact within the state may be less than the local effect, or even negative. However, the state effect often exceeds the national effect. Chhabra et al. [46] noted that festivals are usually a strategic choice for the development of many rural economies but that the economic impact of festivals depends on the characteristics of the festival, such as the number of days the festival is held and the characteristics of the local economy. Other scholars have studied the motivations and purposes behind conference and exhibition consumption [48,49]. However, the forms of these exhibitions (the impacts of conventions and exhibitions on economics) are mainly concerned with transactions; these studies include those by Crompton et al. [50] and Kim et al. [51]. It is a challenge to accurately measure the economic contributions of art exhibitions or exhibition-driven tourism.

### 2.3. Exhibitions and Population 

First, the relationship between population growth and economic development is a problem that has been constantly changing in the field of population economics. This transformation is different in different periods. The most influential document on the relationship between population growth and economic development is “Population”, published by Malthus in 1798, whose findings imply that high community empowerment enables a community to establish successful sustainable tourism development through local people’s support for tourism [52]. For their empirical tests, data were collected from 280 tourists to Japan from South Korea (Nam et al. [16]). The authors found that local people could help enhance tourist destination loyalty from a relational perspective. Hwang and Lee [53] used this study to show that all four dimensions (i.e., education, entertainment, esthetics, and escapism) have a positive influence on well-being perception, which, in turn, positively affects the outcome variables.

Second, as Japan’s society ages, and due to its low fertility rate, city shrinkage has had a tremendously negative effect on the country’s economic development. Over 85% of municipalities experienced population loss from 2005 to 2015 [54]. The shrinking and aging of the Japanese population, coupled with continuous polarization effects towards urban centers, have led to a clear dissolution of the countryside [55,56]. Population problems in the countries near Japan are the same [57], as shown by Hwang et al. [58]. Lastly, extraversion has played a moderating role in the relationship between suitable behavior and activity involvement. Hwang et al. [40] determined the important role of product knowledge as a moderator. Kalwar et al. [59] suggested the development of planning policies to stimulate agricultural industrial development in secondary cities and noted that the devolution of powers can help achieve sustainable development. Although the population has been studied for a long time in various fields, it remains a very important issue.

## 3. The Echigo–Tsumari Art Triennale

### 3.1. From Japanese Art Festivals to ETAT

**Figure 2:**
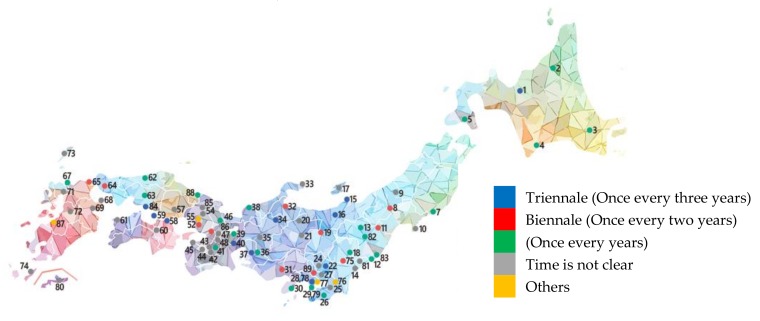
A map of Japanese Art Festivals.

**Figure 3:**
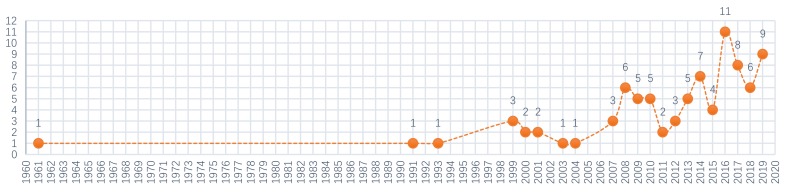
Statistics of the establishment time and number of art exhibitions (1961–2019).

According to the statistics of this article, a total of 88 art exhibitions were created in Japan from 1961 to 2019 (Figure 2). During this period, only seven exhibitions were closed after several sessions. Among them, new art exhibitions began to explode in 2007; 92% of these exhibitions were successful, according to the statistics (Figure 3).

### 3.2. The ETAT

Ever since the 1980s, culture has been recognized as an essential amenity to improve the general quality of life of urban centers and ex-industrial cities [60,61]. Scholars have worked to comprehend the potential of art and culture in remote shrinking contexts [62,63,64]. However, research on this matter is still at an early stage, and more in-depth studies would improve our general understanding of the topic [65].

The ETAT has been described as unique in its quality and scale by media abroad and is highly regarded as a new model for art exhibitions. Community building through art has drawn attention as the ‘Tsumari Approach’ and has been referred to by curators and people in the art industry in the US, Europe, and Asia, as well as by the delegations of local governments, international conferences, and symposia [66]. The ETAT emerged from a prefectural incentive that pushed regions to rely on the specificities of their environments to overcome socio–economic decline [67,68]. 

**Figure 4:**
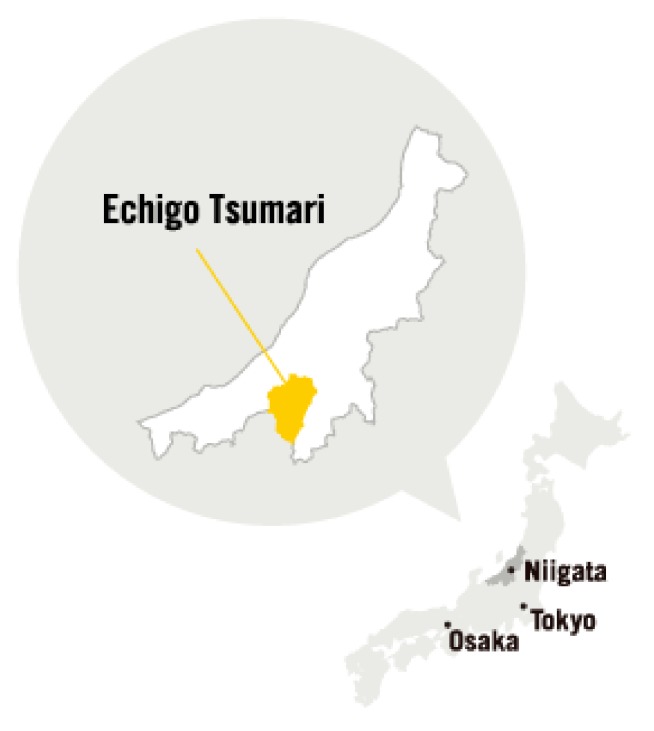
Niigata in Japan.

**Figure 5:**
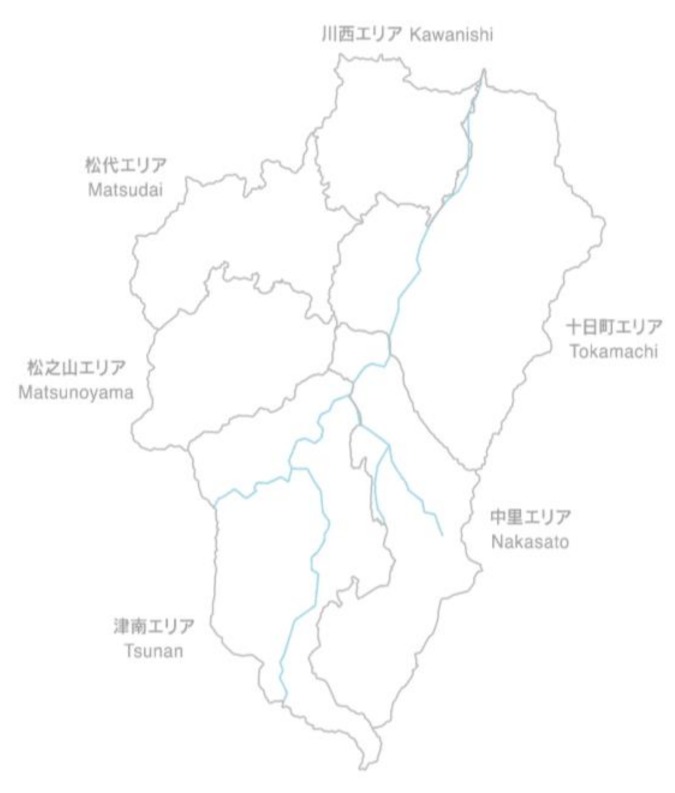
The Echigo–Tsumari Art Triennial (ETAT) areas.

Every three years, artists from all countries are invited to create site-specific pieces of artwork engaging with the specificity of the environmental, social, and cultural context of the Echigo–Tsumari areas (Table 1). Since the first iteration of the ETAT, more than a thousand interventions, including sculptures, sound works, theatrical productions, art installations, performances, musical shows, landscape designs, urban design projects, and architectural constructions, have been dispersed across this 762 km^2^ area. The ETAT areas encompass Tokamachi, Kawanishi, Matsudai, Matsunoyama, Nakasato, and Tsunan, which are all a part of Niigata (Figure 4 and Figure 5). 

**Figure 6:**
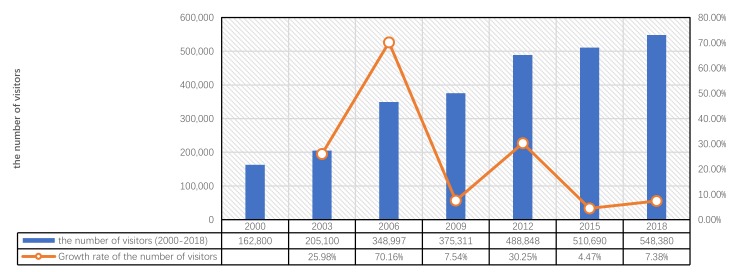
Changes in the number of visitors over the years (2000–2018).

Based on the statistics of the number of visitors from 2000 to 2018, it can be found that: (1) The number of visitors has been increasing every year, indicating that the recognition and influence of the ETAT itself are constantly increasing; (2) based on the annual growth rate, 2006 (with a growth rate of 70.16%) was the fastest-growing year, and the ETAT’s impact on these areas is positive (Figure 6).

Ahn [69] and Maughan [70] studied the impact of the ETAT on the region at the cultural and artistic levels. Klien [4] and Kitagawa [4,71] explored the relationship between man and nature in this exhibition, while Favell [72] and Boven et al. [72,73] researched how the ETAT features abandoned schools and how these schools can help deprecated areas achieve a cultural revival.

According to previous studies, the exhibition (Triennale) is rarely used as a social force to comprehensively evaluate and demonstrate its role in exhibition-driven tourism. Therefore, the current research attempts to fill this gap by empirically investigating the correlation between sustainable tourism, economics, population, and exhibition-driven tourism.

## 4. Methods

### 4.1. Panel Data

Di Lascio et al. [8] studied the relationship between cultural tourism and temporary art exhibitions using a panel data analysis. Panel data are a multi-dimensional type of data involving measurements over time [74]. Panel data contain observations of multiple phenomena obtained over multiple periods for the same objects [75]. Moreover, panel data are more informative than other types of data since they offer more variability and their estimates are, therefore, more efficient [8]. Many studies have used panel data to analyze the impact of tourism on the economy. Naudé and Saayman [76] determined the five main areas important to empirical research on tourism. Many different estimation methods are available [77]. The impacts of climate change on domestic tourism in the UK were studied via panel data estimation [78]. 

In this study, data were collected based on three aspects (number of tourists, per capita income, and household number) (Figure 1), which are explained as follows (Table 2): (1) The areas were divided into two types (the ETAT areas and Niigata). (2) Categorical data included the hosting year of the ETAT (2000/2003/ 2006/2009/2012/2015/2018) (hereafter YES), the years between the hosting of the ETAT (2001/2002/2004/2005/2007/2008/2010/2011/2013/2014/2016/2017) (hereafter BETWEENNESS), and the year before the hosting of the ETAT (1990–1999) (hereafter NO). 

### 4.2. The Descriptive Statistics

These data can be further analyzed effectively through descriptive statistics. Hwang et al. [79] used descriptive statistics to study wellbeing perception and its outcomes in the context of elderly tourism. Other scholars have used descriptive statistics to study the relationship between sustainability and tourism [35,80], while others have used descriptive statistics to study the relationship between economics and tourism [29,33,81]. Shentema et al. [82] used descriptive statistics and Pearson regression for their statistical analyses.

### 4.3. The Correlation Analysis

The panel data were analyzed in two ways (a one-way ANOVA and a Pearson correlation analysis) in SPSS26 Statistics (IBM, New York, NY, USA): (1) The independent variable (the ETAT) was used as a categorical variable (YES-BETWEENNESS-NO). The dependent variables (the corresponding indicators of the ETAT) were used as the continuous variables. (2) The independent variables (the corresponding indicators of the ETAT areas) and the dependent variables (corresponding to the indicators of Niigata) were continuous types.

#### 4.3.1. The One-Way ANOVA Analysis:

A one-way analysis of variance (ANOVA) was used to determine whether there were any statistically significant differences between the means of three or more independent (unrelated) groups [83]. In statistics, a one-way analysis of variance (abbreviated one-way ANOVA) is a technique that can be used to compare the means of two or more samples (using an F distribution) [84]. A one-way ANOVA compares the means between the relevant groups and determines whether any of those means are statistically significantly different from each other. Specifically, it tests the null hypothesis:

(1) $$H_{0}:{µ\;}_{1}={µ\;}_{2}={µ\;}_{3}=\ldots ={µ\;}_{k}$$

where µ is the group mean, and k is the number of groups. If, however, the one-way ANOVA returns a statistically significant result, we accept the alternative hypothesis (HA), which is that there are at least two group means that are statistically significantly different from each other [85].

#### 4.3.2. Pearson Correlation Analysis

A correlation analysis is a statistical method used to evaluate the strength of the relationship between two quantitative variables. A high correlation means that two or more variables have a strong relationship with each other, while a weak correlation means that the variables are hardly related [86,87,88]. In addition, when two random variables (X and Y) are normally distributed, the population’s Pearson product moment correlation coefficient [89] is given by

(2) $$P=\frac{Cov(X,Y)}{\;\sigma x\;\sigma y\;}$$

where σx and σy are the population standard deviations of X and Y, respectively. This coefficient is affected by extreme values and is, therefore, not significant when either or both of the variables are not normally distributed.

## 5. Results

First, we will explain the abbreviations taken from SPSS26 (Table 1, Table 2, Table 3, Table 4, Table 5, Table 6, Table 7, Table 8, Table 9, Table 10, Table 11, Table 12, Table 13 and Table 14): (1) df (degrees of freedom) is the number of independent or freely variable independent variables in the sample. (2) F (F value) means that the larger the F value, the more significant the difference between the variables. (3) Sig. (significance) indicates the significance of the data. If 0.01 < Sig. <0.05, then the difference is significant. If Sig. <0.01, then the difference is extremely significant. (4) N indicates the number of samples.

Next, we will explain the axes (Figure 7, Figure 8, Figure 9, Figure 10, Figure 11, Figure 12, Figure 13, Figure 14, Figure 15, Figure 16 and Figure 17): (1) The X-axis on the left side of the figure represents the number of each variable. (2) The X-axis on the right side of the figure represents the annual rate of change for each variable. (3) The Y-axis below the figure shows the change over time in years.

### 5.1. Number of Tourists

#### 5.1.1. The ETAT and Number of Tourists

**Figure 7:**
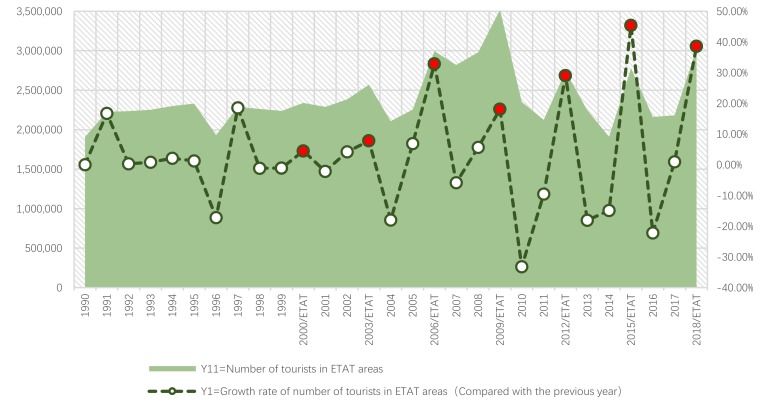
Tourist number and its growth rate in ETAT areas (1990–2018). Note: The red dots in the chart show that the annual growth rate increased (the hosting year of the ETAT).

First, according to the descriptive statistics, the annual change in the number of tourists in ETAT areas (Y11) can be seen intuitively (Figure 7). (1) There is a positive correlation between the tourist number (Y11) and the hosting year of the ETAT (YES). (2) In 2009, the number of tourists reached its highest peak (3,519,310 persons). (3) The maximum annual growth rate occurred in 2015.

**Figure 8:**
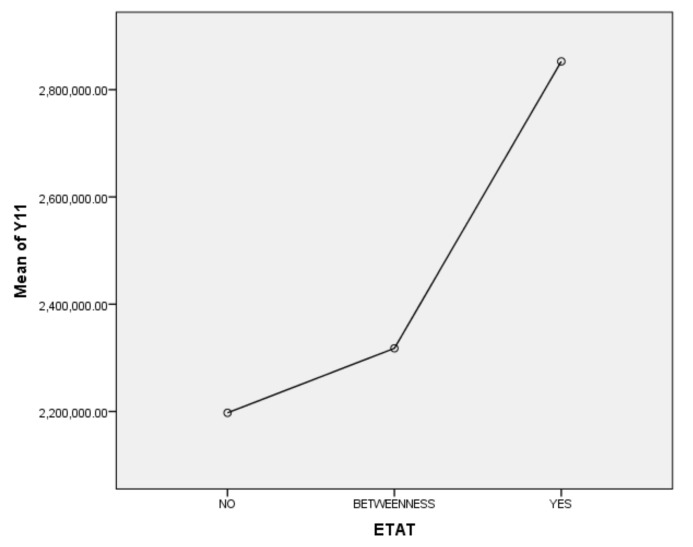
Mean plots.

Second, according to the one-way ANOVA analysis of the tourist numbers of ETAT areas (Y11), (1) Sig. <0.05, which indicates that there is a significant difference between YES, BETWEENNESS, and NO (Table 3). (2) According to the analysis of the difference between YES, BETWEENNESS, NO, and Y11 (Table 4; Table 5; Figure 8), the difference between YES and NO is the largest. The difference between YES and BETWEENNESS is medium, and the difference between BETWEENNESS and NO is the smallest. 

The following conclusions can be summarized: (1) The hosting of the ETAT (YES or NO) has a significant impact on the tourist numbers. (2) The impacts on the tourist numbers during the two years between the hosting of the ETAT are not obvious. (3) This shows that the correlation between hosting the ETAT and the tourist numbers is positive and also illustrates the “rising tide” that exhibition-driven tourism brings to the tourism industry [47].

#### 5.1.2. ETAT Areas and Niigata

**Figure 9:**
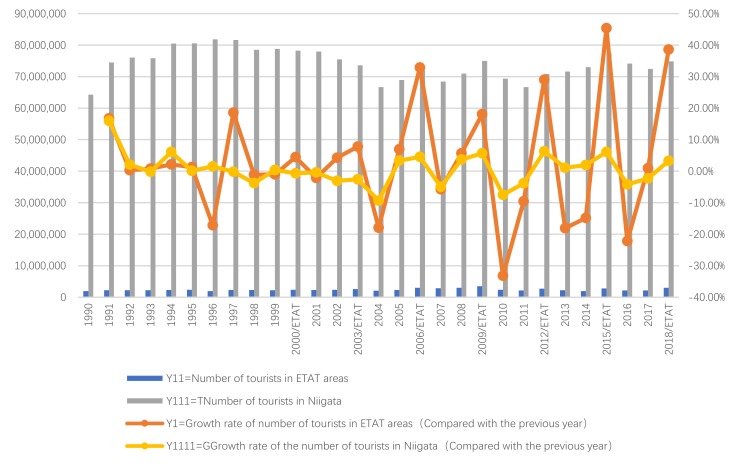
Number of tourists and its growth rate in ETAT areas (1990–2018).

First, according to the descriptive statistics, the annual changes in Y11 and Y111 can be seen intuitively (Figure 9). (1) In terms of quantity, Y111 increased starting in 1990. Its peak occurred in 1996 and 1997. Then, it began to decline; after 2004, it began to fluctuate. There was also a positive relationship between Y11 and Y111. (2) According to the annual growth rate, the change in Y1 was bigger than that in Y1111, but there has been a significant and positive relationship between Y1 and Y1111 since 2000.

**Figure 10:**
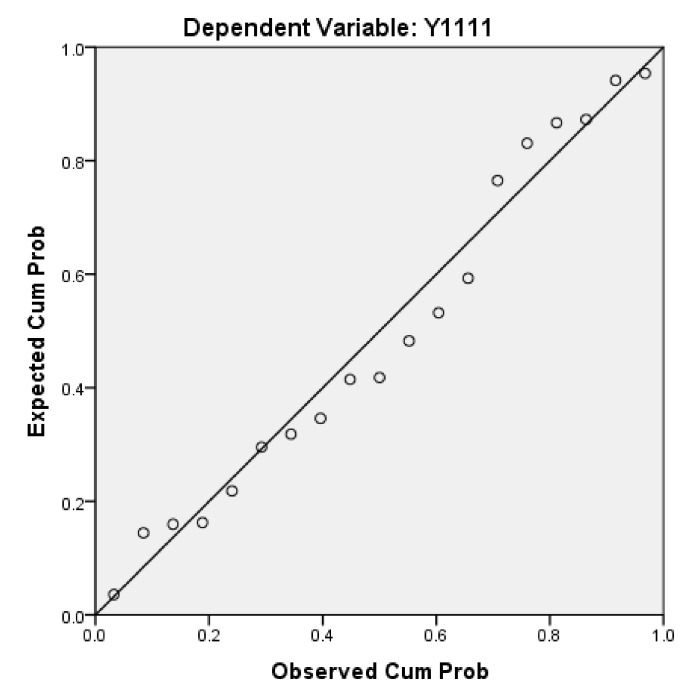
Normal P–P plot regression standardized residual.

Second, according to the Pearson correlation analysis between Y1 and Y1111 (Table 6; Figure 10), (1) the correlation is significant at a 0.01 level (two-tailed), which shows that the correlation model has a high level of credibility. (2) The correlation degree of the Pearson correlation between Y1 and Y1111 is 0.766 (≥0.5). The Pearson Correlation ranges between −1 and +1. If the linear correlation between Y1 and Y1111 is positive (i.e., higher levels of one variable are associated with higher levels of the other) then the results are >0. This indicates that there is a high correlation between Y1 and Y1111. A high correlation means that two or more variables have a strong relationship with each other, while a weak correlation means that the variables are hardly related [89].

In summary, the result shows that (1) before the hosting of the ETAT, there was no particularly strong correlation between Y1 and Y1111. (2) After the EAT was held in 2000, there was a high correlation between Y1 and Y1111.

Based on the above analysis, there is a positive correlation between the ETAT and the tourist numbers. On the other hand, this shows that the ETAT significantly increased the local tourist numbers. Moreover, these effects even exceeded those of the hosting areas. 

### 5.2. Per Capita Income

#### 5.2.1. Between the ETAT and Per Capita Income

**Figure 11:**
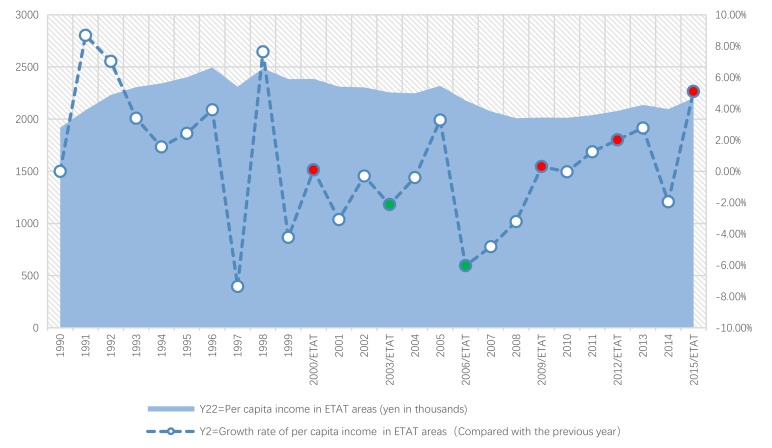
Per capita income and its growth rate in ETAT areas (1990–2018). Note: The red dots in the chart show that the annual growth rate increased (the hosting year of the ETAT). The green dot is negative.

First, according to the descriptive statistics, the annual change of the per capita income of ETAT areas (Y22) can be seen intuitively (Figure 11). (1) There is a positive correlation between Y22 and the hosting year of the ETAT (YES). (2) In 1996 and 1998, Y22 reached its highest peak (3519310 persons). (3) The maximum annual growth rate (Y2) appeared in 2015 (the same as Y1).

**Figure 12:**
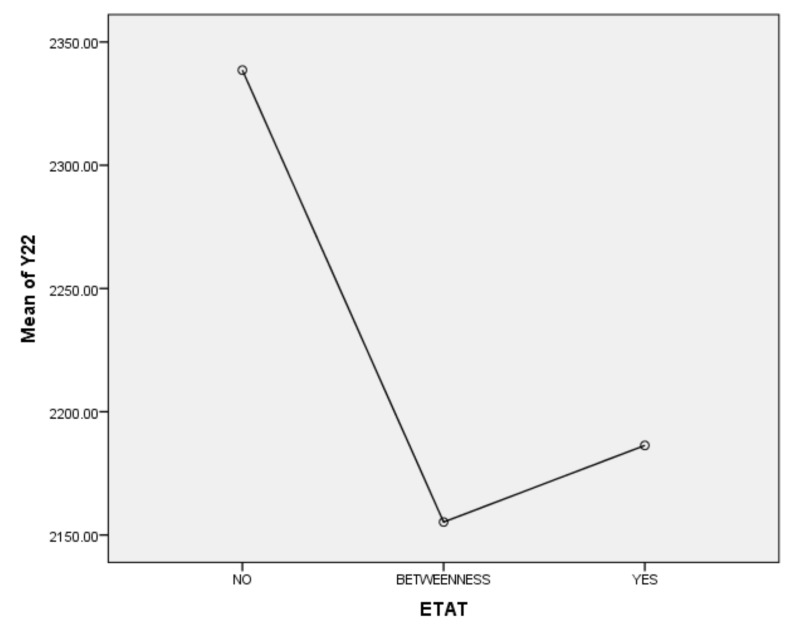
Means Plots.

Second, according to the one-way ANOVA analysis on per capita income in the ETAT areas (Y22) (Table 7), (1) Sig. <0.05, which indicates that there is a significant difference between YES, BETWEENNESS, and NO. (2) According to the analysis of the difference between YES, BETWEENNESS, NO, and Y22 (Table 8; Table 9; Figure 12), the difference between BETWEENNESS and NO is the largest. The difference between YES and NO is medium, and the difference between YES and BETWEENNESS is the smallest.

The following conclusions can thus be summarized: (1) The hosting of the ETAT (YES or NO) has a significant impact on the per capita income. (2) The impacts on the per capita income between the two years of hosting the ETAT are obvious. (3) This shows that the correlation between hosting of the ETAT and per capita income is positive and also illustrates the “rising tide” that exhibition-driven tourism brings to the tourism industry [47].

#### 5.2.2. The ETAT Areas and Niigata

**Figure 13:**
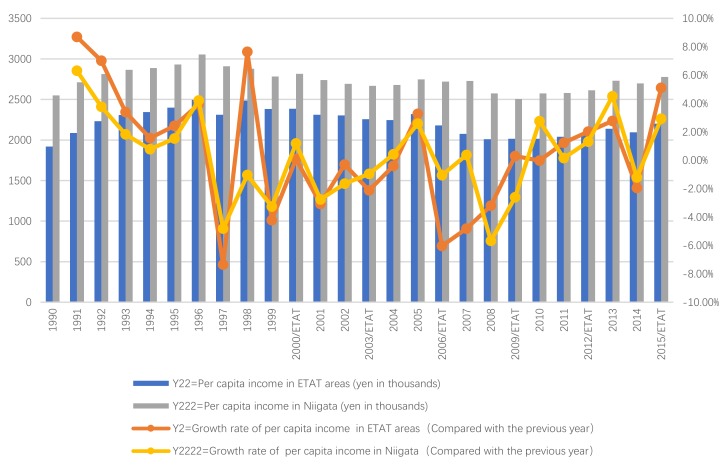
Per capita income and its growth rate in ETAT areas and Niigata (1990–2018).

First, according to the descriptive statistics, the annual changes in per capita income can be seen intuitively (Figure 13). (1) In terms of quantity, the Y222 increased starting in 1990. The peak occurred in 1996. Then, it began to decline; After 2000, it began to fluctuate. There was also a positive relationship between Y22 and Y222. (2) According to the annual growth rate, the change of Y2 was bigger than that of Y2222, but there has been a significant and positive relationship between Y2 and Y2222 since 2000.

**Figure 14:**
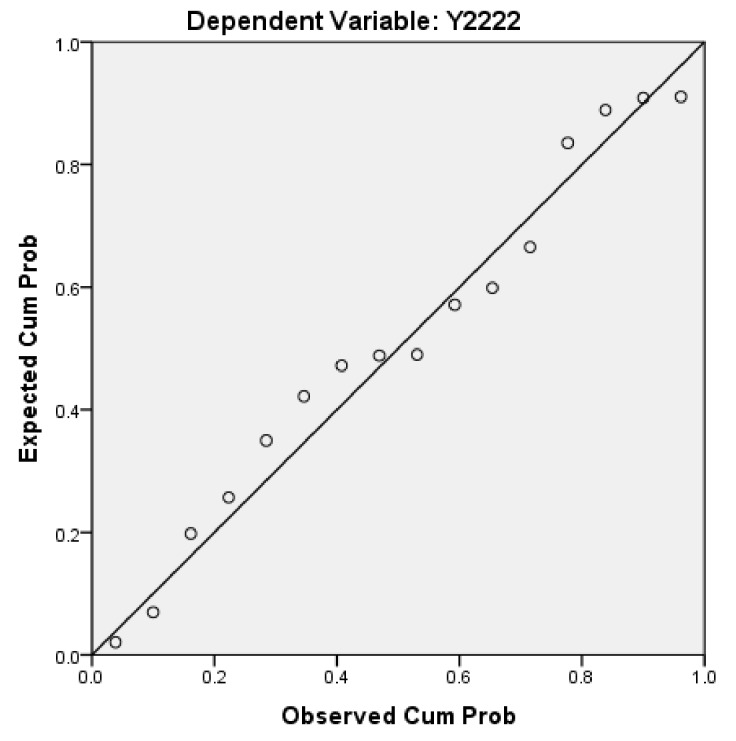
Normal P–P plot regression standardized residuals.

Second, according to the Pearson correlation analysis between Y2 and Y2222 (Table 10; Figure 14), (1) the correlation is significant at a 0.01 level (2-tailed), which shows that the correlation model has a high level of credibility. (2) The correlation degree of the Pearson correlation between Y2 and Y2222 is 0.640 (≥0.5). The Pearson correlation ranges between −1 and +1. If the linear correlation between Y2 and Y2222 is positive (i.e., higher levels of one variable are associated with higher levels of the other) results (>0), then there is a high correlation between Y2 and Y2222 (A high correlation means that two or more variables have a strong relationship with each other, while a weak correlation means that the variables are hardly related) [89].

To sum up, the results show that (1) before the hosting the ETAT, there was no particularly strong correlation between Y2 and Y2222. (2) After the ETAT was held in 2000, There was a strong correlation between Y2 and Y2222.

Based on the above analysis, there is a positive correlation between the ETAT and per capita income. On the other hand, this shows that the ETAT significantly increased local per capita income. Moreover, these effects even exceeded those of the hosting areas.

### 5.3. Household Number

#### 5.3.1. ETAT and Household Number

**Figure 15:**
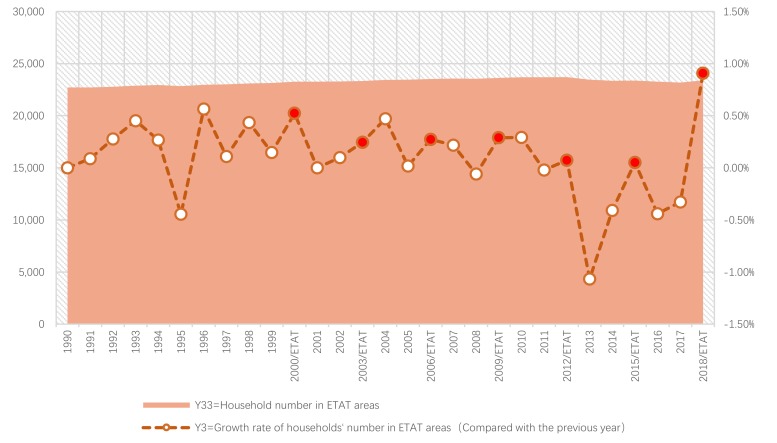
Household number and its growth rate in ETAT areas (1990–2018).

First, according to the descriptive statistics, the annual change of each household number in the ETAT areas (Y33) can be seen intuitively (Figure 15). (1) There is a positive correlation between the Y33 and the hosting year of the ETAT (YES). (2) In 2012, Y33 reached its highest peak (3,519,310 persons). (3) The maximum annual growth rate (Y3) appeared in 2018.

**Figure 16:**
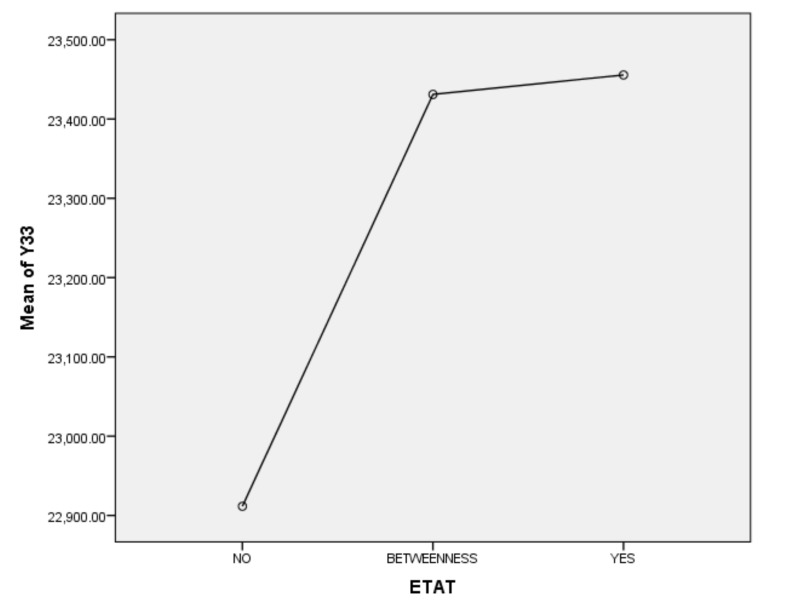
Mean plots.

Second, according to the one-way ANOVA analysis of household numbers in ETAT areas (Y33), (1) Sig. <0.05, which indicates that there is a significant difference between YES, BETWEENNESS, and NO (Table 11). (2) According to the analysis of the difference between YES, BETWEENNESS, NO, and Y33 (Table 12; Table 13; Figure 16), the difference between YES and NO is the largest, while the difference between BETWEENNESS and NO is medium. The difference between YES and BETWEENNESS is the smallest.

We can thus present the following conclusions: (1) The hosting of the ETAT (YES or NO) has a significant impact on the household number. (2) The impacts on the household number during the two years between the hosting of the ETAT are obvious. (3) This shows that the correlation between the hosting of the ETAT and the household number is positive. This also illustrates the “rising tide” that exhibition-driven tourism brings to the tourism industry [47].

#### 5.3.2. Differences between the ETAT areas and Niigata

**Figure 17:**
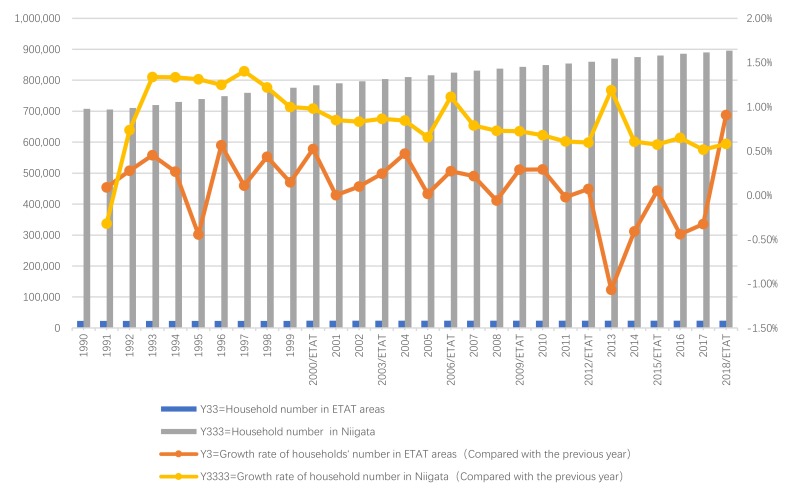
Household number and growth rates in ETAT areas and Niigata (1990–2018).

First, according to the descriptive statistics, the annual changes in household number can be seen intuitively (Figure 17). (1) In terms of quantity, the Y33 and Y333 increased starting in 1990. After 2000, it began to fluctuate. There was no positive relationship between Y33 and Y333. (2) According to the annual growth rate, the change of Y3 was bigger than that of Y3333, but there has been no significant and positive relationship between Y3 and Y3333 since 2000.

Second, according to the Pearson correlation analysis between Y3 and Y3333 (Table 14), (1) the correlation is significant at a 0.529 level (two-tailed) > 0.05 (generally, this is calculated at a confidence level, usually 95% (i.e., the significance level α is equal to 0.05)) [89]. To sum up, the results show that there was no particularly strong correlation between Y3 and Y3333.

Therefore, based on the above analysis, there is a positive correlation between the ETAT and household number. On the other hand, this analysis shows that the ETAT significantly increased the local per capita income, but no effects exceeded those of the hosting areas.

## 6. Discussion

### 6.1. Impactions

The logical model was developed based on a comprehensive literature review and empirical evidence. The question (did these art exhibitions have some positive sustainable impacts on the hosting areas?) should be rethought. If we were to do nothing to declining rural areas, then everything in those areas would disappear (including all the green and beautiful environments). Even if we one day desired to return to nature, it would be extremely difficult to fix the environment destroyed by humans. Indeed, even if we were involved in exhibition-driven tourism, such as the ETAT, we would not be able to pull these areas out of their recessions and allow them to grow positively. However, if we try to maintain a sustainable environment based on exhibition-driven tourism, we can have positive impacts on these areas. The present study suggests that this model (exhibition-driven tourism) can be successfully used in Japan and other areas (with similar conditions) as a sustainable form of tourism (and an economic green model) that can also positively affect the local population.

### 6.2. Limitations and Future Research

First, the total population and labor force (15–65 years old) in the entire area are still declining [90]. The Triennale has not halted this negative phenomenon [91]. However, we find that decreases in the total population and labor force in other rural areas (and even urban areas) are a common phenomenon across Japan [18]. Thus, the positive changes in household number represent a positive impact of exhibition-driven tourism (the ETAT) in these areas (although there are many influences behind the number of families).

Second, based on the above analysis, there is a positive correlation between the ETAT and the three studied aspects (sustainable tourism, economics, and population). Further, the results show that the ETAT significantly increased local sustainable tourism, economics, and the population. These effects even exceeded those of the hosting areas. However, for the population, the impact of the hosting area on the larger area is not obvious. 

Third, this article has studied the positive impacts of exhibition-driven tourism (the ETAT) based on three aspects (sustainable tourism, economics, and population) only. The data on these aspects may be affected by some other social and economic events. However, the result from the data analysis provides evidence on the impacts of the relationship between exhibition-driven tourism (the ETAT) and the three studied aspects. Thus, we suggest that researchers in other parts of Japan and on other continents work together to produce similar studies, thereby creating a worldwide body of literature examining the phenomena related to the effects of certain types of festivals on key community variables [52,58]. 

Finally, the current study employed some nonprobability approaches, such as descriptive statistics and a correlation analysis (a one-way ANOVA and a Pearson correlation analysis in SPSS26). Even though these methods are widely used in the tourism industry, it is difficult to represent the overall impacts of exhibition-driven tourism. Hence, future studies should use a greater sampling range.

## 7. Conclusions

Exhibition-driven tourism will bring new opportunities to the tourism sector. Tourism is one of the fastest-growing industries and a driving force for many developed and developing economies [92]. In particular, exhibition-driven tourism prefers sustainable tourism to ensure a green experience during visits. Furthermore, exhibition-driven tourism has been spotlighted in the tourism context, as more tourists are concerned with art exhibitions. This equally applies to sustainable economics and the population. Despite the tremendous opportunities of exhibition-driven tourism (i.e., the growing demands for this type of tourism), the existing literature on the tourism industry has offered limited research pertaining to exhibition-driven tourism in association with its positive impacts (for sustainable tourism, economics, and the population). That is to say, little is known about the intricate relationships between sustainable tourism, economics, and the population and exhibition-driven tourism. In addition, no attempt has been made to investigate the moderating effect of the ETAT in the links among these impacts in a tourism context. The present study successfully fills this void and considers the roles of exhibition-driven tourism in such relationships. The present findings thus offer meaningful implications in both academia and industry.

## Figures and Tables

**Table 1:** Some concepts of the ETAT.

Concept	Main Contents
Humans are part of nature	As our civilization reaches its critical juncture, the rich nature of Satoyama’s presence in Echigo–Tsumari can impel us to review our attitudes toward the environment, calling into question the modern paradigms that have caused so much environmental destruction.
Satoyama and Art	The nature and lifestyle of the Satoyama in the Echigo–Tsumari environment seems to inspire artists to recover the connections and collaborations that art once had but have almost been lost.
Cooperation beyond generations, regions, and genre	The artists’ passion and openness to learning inspires local people who engage with the artwork, not as spectators, but as collaborators.
Festival	In addition to the Triennale itself, visitors can enjoy the Summer Festival (“Daichi-no-matsuri”) and the winter “Snow Art Project”, which coincide with local festivals and traditional events throughout the year. The “Daichi-no-matsuri” takes place in the years between the Triennale, welcoming visitors and opening various works of art to the public.
Events and performances	Performances and entertainment from all over the world are presented on the unique stage of Echigo–Tsumari set against the area’s artwork and terraced rice fields. Visitors can enjoy local expressions and entertainment.

**Table 2:** Observed variables: Name, type, and data source.

Variables	Name	Type	Sources
X YES BETWEENNESS NO	The ETAT the hosting year of the ETAT the year between the hosting of the ETAT the year before the hosting of the ETAT	Categorical ^1^	ETAT Official website
Y1	Growth rate of number of tourists in ETAT areas ^3^	Continuous	Our elaborations on NSY ^2^ data sets
Y11	Number of tourists in ETAT areas		
Y111	Number of tourists in Niigata		
Y1111	Growth rate of the number of tourists in Niigata ^3^		
Y2	Growth rate of the per capita income in ETAT areas ^3^	Continuous	Our elaborations on NSY data sets
Y22	Per capita income of ETAT areas (yen in thousands)		
Y222	Per capita income of Niigata (yen in thousands)		
Y2222	Growth rate of the per capita income in Niigata ^3^		
Y3	Growth rate of household number in ETAT areas ^3^	Continuous	Our elaborations on NSY data sets
Y33	Household number in ETAT areas		
Y333	Household number in Niigata		
Y3333	Growth rate of household number in Niigata ^3^		

^1^ Categorical (YES/BETWEENNESS/NO); ^2^ NSY = Niigata Statistical Yearbook; ^3^ Compared with the previous year.

**Table 3:** One-way ANOVA (Y11).

	Sum of Squares	df	Mean Square	F	Sig.
Between Groups	1,924,458,691,410	2	962,229,345,705	12.180	0.000
Within Groups	2,054,037,748,901	26	79,001,451,880		
Total	3,978,496,440,312	28			

**Table 4:** One-way descriptive statistics (Y11).

Variables	N	Mean	Std. Deviation	Std. Error	95% Confidence Interval for Mean		Minimum	Maximum
					Lower Bound	Upper Bound		
NO	10	2,197,479	150,206	47,499	2,090,027	2,304,930	1,908,400	2,330,450
BETWEENNESS	12	2,317,784	300,873	86,854	2,126,618	2,508,950	19,114,950	2,979,990
YES	7	2,852,618	377,537	142,695	2,503,454	3,201,781	23,386,700	3,519,210
Total	29	2,405,397	376,947	69,997	2,262,014	2,548,780	19,084,000	3,519,210

**Table 5:** Post hoc tests: Multiple comparisons (Y11).

(I) ETAT	(J) ETAT	Mean Difference (I–J)	Std. Error	Sig.	95% Confidence Interval	
					Lower Bound	Upper Bound
NO	BETWEENNESS	−120,305	120,347	0.327	−367,684	127,072
	YES	−655,139 *	138,513	0.000	−939,858	−370,420
BETWEENNESS	NO	120,305	120,347	0.327	−127,072	367,684
	YES	−534,833 *	133,676	0.000	−809,609	−260,057
YES	NO	655,139 *	138,513	0.000	370,420	939,858
	BETWEENNESS	534,833 *	133,676	0.000	260,057	809,609

* The mean difference is significant at a 0.05 level.

**Table 6:** Correlations between Y1 and Y1111.

		Y1	Y1111
Y1	Pearson Correlation	1	0.766 **
	Sig. (two-tailed)		0.000
	N	19	19
Y1111	Pearson Correlation	0.766 **	1
	Sig. (two-tailed)	0.000	
	N	19	19

** Correlation is significant at a 0.01 level (2-tailed).

**Table 7:** One way ANOVA (Y22).

	Sum of Squares	df	Mean Square	F	Sig.
Between Groups	173,367	2	86,683	5.259	0.014
Within Groups	362,624	22	16,482		
Total	535,991	24			

**Table 8:** Oneway descriptive statistics (Y22).

Variables	N	Mean	Std. Deviation	Std. Error	95% Confidence Interval for Mean		Minimum	Maximum
					Lower Bound	Upper Bound		
NO	9	2338	127.37473	42.45824	2240.6644	2436.4822	2085.49	2495.12
BETWEENNESS	10	2155	127.98682	40.47298	2063.6937	2246.8063	2008.50	2320.00
YES	6	2186	130.69341	53.35536	2049.1790	2323.4877	2014.50	2385.00
Total	25	2228	149.44229	29.88846	2167.0196	2290.3931	2008.50	2495.12

**Table 9:** Post Hoc Tests: Multiple Comparisons (Y22).

(I) ETAT	(J) ETAT	Mean Difference(I–J)	Std. Error	Sig.	95% Confidence Interval	
					Lower Bound	Upper Bound
NO	BETWEENNESS	183 *	58	0.005	60	305
	YES	152 *	67	0.035	11	292
BETWEENNESS	NO	−183 *	58	0.005	−305	−60
	YES	−31	66	0.644	−168	106
YES	NO	−152 *	67	0.035	−292	−11
	BETWEENNESS	31	66	0.644	−106	168

* The mean difference is significant at a 0.05 level.

**Table 10:** Correlations between Y2 and Y2222.

		Y2	Y2222
Y2	Pearson Correlation	1	0.640 **
	Sig. (2-tailed)		0.008
	N	16	16
Y2222	Pearson Correlation	0.640 **	1
	Sig. (2-tailed)	0.008	
	N	16	16

** Correlation is significant at a 0.01 level (2-tailed).

**Table 11:** One-way ANOVA (Y33).

	Sum of Squares	df	Mean Square	F	Sig.
Between Groups	1,832,318	2	916,159	35.584	0.000
Within Groups	669,412	26	25,746		
Total	2,501,730	28			

**Table 12:** One-way descriptive statistics (Y33).

Variables	N	Mean	Std. Deviation	Std. Error	95% Confidence Interval for Mean		Minimum	Maximum
					Lower Bound	Upper Bound		
NO	10	22,911	149	47	22,804	23,018	22,703	23,136
BETWEENNESS	12	23,431	168	48	23,323	23,538	23,185	23,689
YES	7	23,455	161	61	23,306	23,604	23,257	23,701
Total	29	23,257	298	55	23,144	23,371	22,703	23,701

**Table 13:** Post hoc tests: Multiple comparisons (Y22).

(I) ETAT	(J) ETAT	Mean Difference (I–J)	Std. Error	Sig.	95% Confidence Interval	
					Lower Bound	Upper Bound
NO	BETWEENNESS	−519 *	68	0.000	−660	−378
	YES	−543 *	79	0.000	−706	−381
BETWEENNESS	NO	519 *	68	0.000	378	660
	YES	−24	76	0.750	−181	132
YES	NO	543 *	79	0.000	381	706
	BETWEENNESS	24	76	0.750	−132	181

* The mean difference is significant at a 0.05 level.

**Table 14:** Correlations between Y3 and Y3333.

		Y3	Y3333
Y3	Pearson Correlation	1	−0.131
	Sig. (two-tailed)		0.592
	N	19	19
Y3333	Pearson Correlation	−0.131	1
	Sig. two-tailed)	0.592	
	N	19	19
